# The Coupling Between Cell Wall Integrity Mediated by MAPK Kinases and SsFkh1 Is Involved in Sclerotia Formation and Pathogenicity of *Sclerotinia sclerotiorum*

**DOI:** 10.3389/fmicb.2022.816091

**Published:** 2022-04-25

**Authors:** Jie Cong, Kunqin Xiao, Wenli Jiao, Cheng Zhang, Xianghui Zhang, Jinliang Liu, Yanhua Zhang, Hongyu Pan

**Affiliations:** ^1^College of Plant Sciences, Jilin University, Changchun, China; ^2^College of Resource and Environment, Jilin Agricultural University, Changchun, China

**Keywords:** *Sclerotinia sclerotiorum*, SsFkh1, MAPK, CWI, sclerotia, pathogenicity

## Abstract

The plant pathogenic fungus *Sclerotinia sclerotiorum* can survive on a wide range of hosts and cause significant losses on crop yields. FKH, a forkhead box (FOX)-containing protein, functions to regulate transcription and signal transduction. As a transcription factor (TF) with multiple biological functions in eukaryotic organisms, little research has been done on the role of FKH protein in pathogenic fungi. *Ss*Fkh1 encodes a protein which has been predicted to contain FOX domain in *S. sclerotiorum*. In this study, the deletion mutant of SsFkh1 resulted in severe defects in hyphal development, virulence, and sclerotia formation. Moreover, knockout of *SsFkh1* lead to gene functional enrichment in mitogen-activated protein kinase (MAPK) signaling pathway in transcriptome analysis and SsFkh1 was found to be involved in the maintenance of the cell wall integrity (CWI) and the MAPK signaling pathway. Yeast two-hybrid and bimolecular fluorescence complementation assays showed that SsFkh1 interacts with SsMkk1. In addition, we explored the conserved MAPK signaling pathway components, including Bck1, Mkk1, Pkc1, and Smk3 in *S. sclerotiorum*. Δ*Ssmkk1*, Δ*Sspkc1*, Δ*Ssbck1*, and Δ*Sssmk3*knockout mutant strains together with Δ*Ssmkk1*^com^, Δ*Sspkc1*^com^, Δ*Ssbck1*^com^, and Δ*Sssmk3*^com^ complementation mutant strains were obtained. The results indicated that Δ*Ssmkk1*, Δ*Sspkc1*, Δ*Ssbck1*, and Δ*Sssmk3* displayed similar phenotypes to Δ*Ssfkh1* in sclerotia formation, compound appressorium development, and pathogenicity. Taken together, SsFkh1 may be the downstream substrate of SsMkk1 and involved in sclerotia formation, compound appressorium development, and pathogenicity in *S. sclerotiorum*.

## Introduction

*Sclerotinia sclerotiorum* is a common phytopathogen that affects a broad range of the plant species. It is one of the most damaging pathogens causing extensive damage to soybean, sunflower, and rapeseed, resulting in large amount of losses in each year (Guyon et al., 2014). Furthermore, *S. sclerotiorum* produces dormant melanized and long-lived multicellular structures, sclerotia, which make it difficult to defense this fungus using fungicides in the field (Zhu et al., 2013).

Sclerotia are asexual dormant structure composed of concentrated vegetative hyphal cells, which are produced by a series of plant pathogenic fungi. These structures contribute to the survival of fungi in harsh environments, such as low temperature, microbial infection, or lack of nutritional host (Smith et al., 2015). *Sclerotinia sclerotiorum* produce sclerotia to survive for long terms under adverse circumstances (Bolton et al., 2010). Sclerotia could germinate infectious hyphae, infecting the stems or roots of the plant directly, or to germinate apothecia, which release ascospores to air as the primary infection source in sclerotinia diseases (Guogen et al., 2017). Sclerotia development is a crucial biological process (BP) in the life of *S. sclerotiorum*. Environmental changes and metabolisms affect sclerotia development (Erental et al., 2008). In addition, sclerotia development was regulated by cyclic adenosine monophosphate (cAMP)-dependent protein kinase, extracellular signal-regulated kinase (ERK)-like mitogen-activated protein kinase (MAPK), transcription factor (TF) SsC6TF1 and secreted protein Sscaf1 (Fan et al., 2017). Furthermore, the melanin in sclerotia is important for *S. sclerotiorum* to overcome the unfavorable environment. However, the molecular regulation mechanisms of sclerotia development in *S. sclerotiorum* are still unknown.

The forkhead box (FOX) TF contain a wing-like helix structure in DNA-binding region, a chromatin conformational change region, a nuclear localization region, and a transcription effector region (Takatani et al., 2004). These transcription factors show functionality in normal growth and development, cell differentiation, metabolism, and other BPs (Carlsson and Mahlapuu, 2002; Arsenault et al., 2015). FOX TFs are highly conserved in fungal and animal genomes. In *Saccharomyces cerevisiae*, FKH1, FKH2, FHL1, and HCM1 were reported (Postnikoff et al., 2012; Arsenault et al., 2015; Pataki et al., 2017). The absence of *MoFKH1* in *Magnaporthe oryzae* impaired conidial germination, mycelial growth, and virulence (Park et al., 2014). In *Candida albicans*, CaFKH2 controls the morphogenesis of fungal hyphae and pseudohyphal toxicity (Bensen et al., 2002). In this study, the deletion of SsFkh1 affects sclerotia development and pathogenicity. However, the regulation mechanism of SsFkh1 regulating the sclerotia development and infection cushion in *S. sclerotiorum* is unclear.

Mitogen-activated protein kinase signaling pathways are ubiquitous in eukaryotes and are involved in cellular development, differentiation, stress, and nutrition, among other processes. In *S. cerevisiae*, MAPK pathways have been found to be involved in the cell wall integrity (CWI) pathway (Gu et al., 2015). The CWI pathway is important for the highly controlled and polarized restructuring of the fungal cell walls during development, morphological changes, and the environmental challenges (Fuchs and Mylonakis, 2009). CWI is activated by cell surface sensors, Wsc1, Wsc2, Wsc3, Mid2, and Mtl1. In *S. cerevisiae*, during cell wall stress, Wsc1 activates the small G protein Rho1 through the guanosine nucleotide exchange factor Rom2. Then Rho1 activates Pkc1 (the protein kinase C), which then phosphorylates the upstream kinase of conserved MAPK cascade Bck1. Bck1, in turn, transmits the signal to the redundant pair of MAP kinase kinases Mkk1 and Mkk2, then MAPK Slt2/Mpk1 was phosphorylated and activated. Finally, Slt2/Mpk1 phosphorylates Rlm1 transcription factor and SBF complex to regulate gene expression (Levin, 2005, 2011; Jendretzki et al., 2011; Sanz et al., 2012, 2016). The CWI pathway is largely conserved in other fungi. The homologs of Bck1 and Slt2/Mpk1, MoMck1, and MoMps1 in *M. oryzae*, respectively, were crucial for pathogenicity and CWI (Jeon et al., 2008). Surprisingly, the functions of MAPK cascade in CWI of *S. sclerotiorum* are not known.

In this study, SsFkh1 regulates sclerotium and compound appressorium (infection cushion) development, and responses to cell wall sensitivity. This study revealed the involvement of SsFkh1 in maintenance of cell wall integrity mediated by MAPK kinases. We investigated the function of conserved MAPK signaling pathway components, including Bck1, Mkk1, Pkc1, and Smk3, regulate sclerotia formation, the development of the compound appressorium, and the CWI pathway in *S. sclerotiorum*. We also specifically elucidated the relationships between SsFkh1 and SsMkk1. SsFkh1 may be involved in cell wall integrity pathway mediated by MAPK kinases, which regulated sclerotia formation and pathogenicity in *S. sclerotiorum*.

## Materials and Methods

### Fungal Strains, Plant Materials, and Culture Conditions

*Sclerotinia sclerotiorum* 1980 (UF-70) wild-type strain was used for gene deletion and complementation. All strains were grown on potato dextrose agar (PDA) at 25°C. Plant materials, including cowpea, tomatoes, and *Arabidopsis* were cultivated at 22°C with 16 h light period (Liu et al., 2018).

### Construction of Mutants and PCR Verification of Gene Deletion

All PCR and quantitative reverse transcription PCR (qRT-PCR) primer sequences used in this study are shown in Supplementary Table S1.

*SsFkh1* (SS1G_07360) gene were deleted with homologous recombination. The 5′ region (~1.5 kb) and the 3′ region (~1.5 kb) of the *SsFkh1* gene were amplified from the genomic DNA (gDNA) of WT *S. sclerotiorum* and were cloned into the pXEH vector to generate a pXEH-L-R construct containing a hygromycin phosphotransferase (hph) cassette driven by a *trpC* promoter (Wang et al., 2016). The successfully constructed plasmids used for PEG-mediated protoplasts transformation to improve the efficiency of homologous recombination. The hyphal tips growing on the selection medium containing 600 μg/ml hygromycin B were transferred onto PDA plates (including 100 μg/ml hygromycin B) and subsequently regenerated five more times on PDA containing 100 μg/ml hygromycin B with 5 days intervals. Hygromycin-resistant transformants obtained by protoplast transformation were verified by PCR (Rollins, 2003).

*SsMkk1* (SS1G_00059), *SsBck1* (SS1G_10983), *SsSmk3* (SS1G_05445), and *SsPkc1* (SS1G_14026) genes were deleted with CRISPR-Cas9 system. The CRISPR-Cas9-TrpC-Hyg vector was used for single guide RNA (sgRNA) construction as previously described (Hisano et al., 2015). The *SsMkk1*, *SsBck1*, *SsSmk3*, and *SsPkc1* genes were selected as the initial mutagenesis targets, respectively. The sgRNA primers for target sites within the *SsMkk1*, *SsBck1*, *SsSmk3*, and *SsPkc1* locus were designed using the online E-Crispr tool, respectively. CRISPR-Cas9-mediated insertion site sequences were identified by thermal asymmetrical interlaced PCR (TAIL-PCR). Each sgRNA primers (M-F/MR, B-F/BR, S-F/SR, and P-F/PR) used in TAIL-PCR was 100 μM, T4 PNK used for phosphorylating and annealing the sgRNA oligos (Phosphorylate and anneal the oligos using the following parameters: 37°C for 30 min; 95°C for 5 min; ramp down to 25°C at 5°C min^−1^). Dilute phosphorylated and annealed oligos 1:50 by adding 1 μl of product from the initial PCR to 49 μl of room temperature ddH_2_O. Cloning the sgRNA oligos into CRISPR-Cas9-TrpC-Hyg vector and transforming it into *Escherichia coli* DH*5α* (Liu and Chen, 2007). The final TAIL-PCR products were purified from an 0.8% agarose gel using the TIANgel Midi Purification Kit (TIANGEN, China, catalog no. DP209-02) and then send out for sequencing. For 3′ sequence insertion characterization, the specific primer was used for each reaction. The successfully constructed plasmids used for PEG-mediated protoplasts transformation. The screening of transformants and verification was performed as mentioned above.

### Construction of Complementation Strains and PCR Verification

For genetic complementation of *SsFkh1* deletion mutants, a genomic region containing the full-length fragment of SsFkh1, including upstream and downstream of the coding sequence, was cloned from the WT *S. sclerotiorum* gDNA. This fragment was then cloned into pD-NEO1 vector carrying the promoter *trpC* (Wang et al., 2016). The complementation transformants were screened on PDA medium with 100 μg/ml G418 and then verified by PCR. The vector pYf-11 was used to genetic complementation of *SsMkk1*, *SsBck1*, *SsSmk3*, and *SsPkc1* deletion mutants. The transformants on G418 selected medium were screened by protoplast transformation and subsequently regenerated five more times on PDA containing 100 μg/ml G418 with 5 days intervals. The transformants were identified by PCR.

### Phenotypic Analysis

For colony morphology, 5 mm diameter mycelial plugs cut from the edge of colonies grown for 2 days of each strain were placed on PDA medium and incubated at 25°C. After 3 days, colony diameters of each strain were measured and recorded. Each experiment included three replicates and was repeated three times. After 14 days, the number and dry weight of sclerotia were determined. GraphPad prism was used for *t*-tests and multiple *t*-tests to analyze significant differences. All analyses in this study used the same statistical methods. For quantification of compound appressoria assays, 5 mm diameter fresh mycelial plugs were inoculated on glass slides and then incubated under suitable humidity conditions at 25°C for 3 days. For morphological observation of compound appressoria, 5 mm diameter fresh mycelial plugs were placed on the glass slide and were placed in a box at 25°Cfor 24 h. The optical microscope was used to observed compound appressoria. For oxalic acid (OA) production assay, the strains were grown on PDA medium supplemented with 100 μg/ml bromophenol and pH adjusted to 7.0 using NaOH, incubated at 25°C in the dark. Color changes were recorded at 2 dpi.

### Response to Stress

To investigate sensitivities to different stresses, mycelial plugs of each strain were inoculated onto PDA added with different agents, which included H_2_O_2_ (15 mM), Calcium fluoride white (CFW, 0.5 mg/ml), and Congo red (CR, 150 mg/ml). Response to stress was observed, and colony diameters were determined on day 3. Three replicates were conducted for each treatment.

### RNA Extraction and Quantitative Real-Time PCR Analysis

Total RNA was extracted from hypha harvested from the strain grown on PDA plates for 2 days. Each hypha sample (0.1 g) was ground into a fine powder in liquid nitrogen, using a TransZol Up Plus RNA kit (TransGen Biotech Co., Ltd., Beijing, China). Then, the resulting product was reverse-transcribed into cDNAs (TransScript All-in-One First-Strand cDNA Synthesis SuperMix for qPCR, TransGen Biotech Co., Ltd., Beijing, China). Quantitative Real-Time PCR was used to analyze the expression levels of *SsFkh1*, *SsMkk1*, *SsBck1*, *SsSmk3*, and *SsPkc1* with a PrimePro 48 real-time detection system (TECHNE, United Kingodm). The actin gene was used as an endogenous reference, and transcript levels were calculated by the 2^–△△Ct^ method (Zhang et al., 2021). Each experiment was repeated three times.

### Pathogenicity Assay

For pathogenicity assays, 5 mm diameter mycelial plugs cut from the edge of colonies grown for 2 days of WT, Δ*Ssfkh1*, and Δ*Ssfkh1^com^* strains were inoculated on cowpea leaves. The mycelial plugs cut from the edge of colonies grown for 2 days of WT, Δ*Ssmkk1*, Δ*Sspkc1*, Δ*Ssbck1*, and Δ*Sssmk3* strains were inoculated on wounded and unwounded tomato leaves. The inoculated leaves were placed in chamber and cultivated at 25°C for 3 days.

### Transcriptomic Analysis

RNA-Secq using DESeq2 (version 1.12.4), Q-value transcripts <0.05 and |log2 (fold change)| > 1 was used. The DEGs were detected and the biological information analysis of DEGs was carried out as previously reported (Hou et al., 2020). The enrichment analysis of genes expressed differentially (GO) was conducted using top GOs that assigned every DEG to a single GO keyword. In the DEGs, the GO keywords in their corrected Q-value of 0,05 were regarded as highly enriched. A global categorization method for gene function contained a database of three different ontological biological processes, cellular components (CC), and molecular functions (MF) that were employed in the analysis^1^ and KEGG.^2^

### Yeast Two-Hybrid Assay

Yeast two-hybrid (Y2H) was used to determine the possible protein–protein interactions between *SsFkh1* and *SsMkk1*. Bait plasmid was constructed by cloning the amplified cDNA fragment of *SsFkh1* with primer pairs 1F/1R into the vector pGBKT7. The full-length cDNA of *SsMkk1* was amplified with primer pairs 2F/2R. The resulting fragments were cloned into the vector pGADT7 to generate prey plasmids. Both bait and prey plasmids were determined by sequencing and then co-transformed into yeast strain (AH109) using a lithium acetate transformation protocol. The transformants growing on SD-Trp/Leu solid medium were isolated and determined on SD-Trp/Leu/His and SD-Trp/Leu/His/Ade solid medium. The addition of X-α-gal was used to assess the activity of α-galactosidase by the production of blue precipitate. The interaction between pGADT7-T and pGBKT7-53 were used as positive control, and between pGBKT7-Lam and pGADT7-T were used negative control, respectively (Zhu et al., 2019).

### Bimolecular Fluorescence Complementation Assay

Bimolecular fluorescence complementation (BiFC) assay was performed in an *Arabidopsis* protoplast system to confirm protein interaction (Fan et al., 2008). SsFkh1 and SsMkk1 were fused with separate regions of yellow fluorescent protein (YFP; 2005) to generate YFP-N-SsFkh1 and YFP-C-SsMkk1 for *Arabidopsis* protoplast transfection (Yoo et al., 2007). The fluorescent signal and localization of fusion proteins were detected using an inverted fluorescence microscope (Eclipse Ts2R; Nikon, NY, United States; emission, 514 nm). The nucleus was visualized by DAPI (4′,6-diamidino-2-phenylindole).

## Results

### Deletion of SsFkh1 Affects Sclerotia Development and Pathogenicity

**Figure 1:**
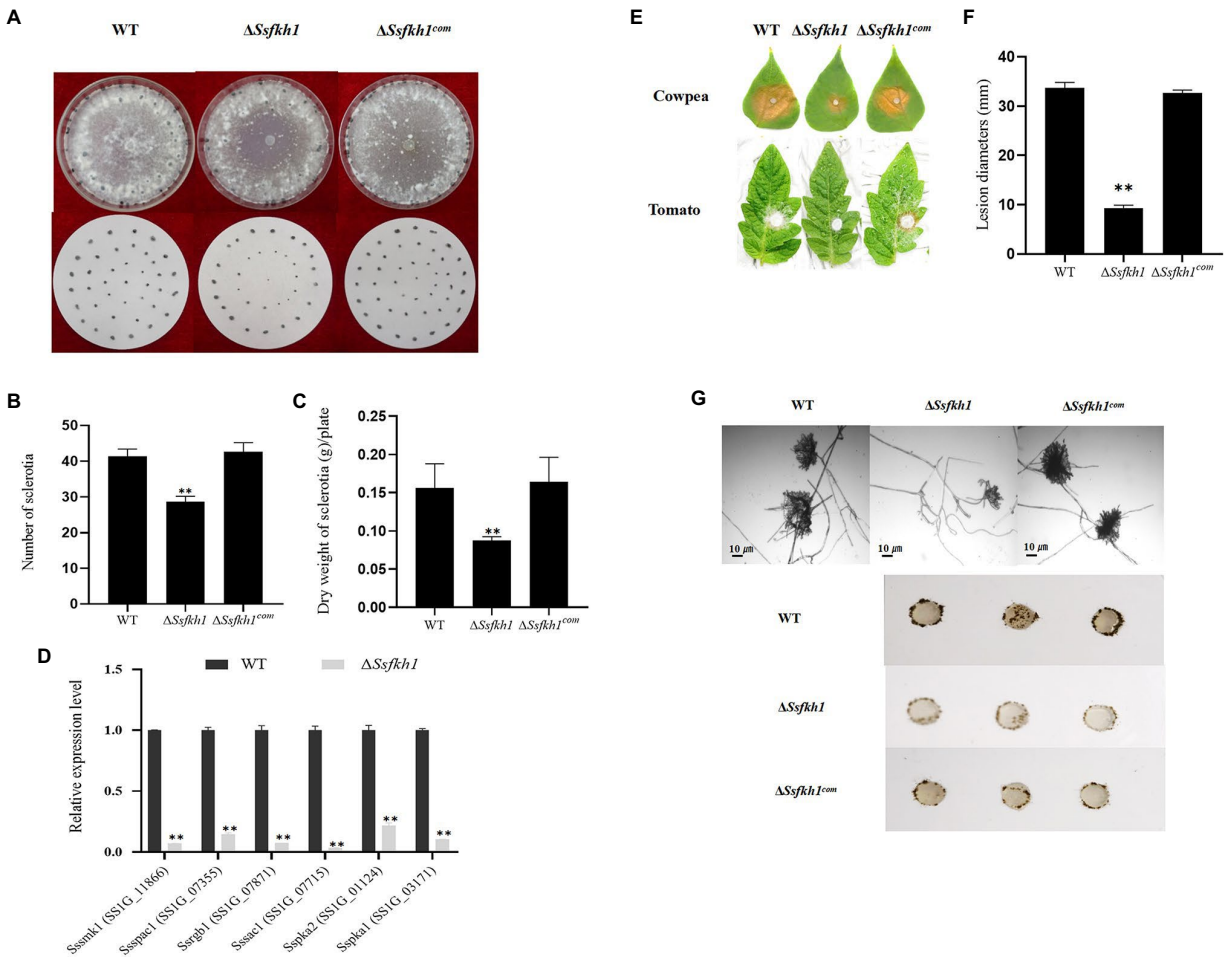
**(A)** Morphologic observation of the WT, Δ*Ssfkh1*, and Δ*Ssfkh1^com^* strains on potato dextrose agar (PDA) medium. Photographs were taken by 14 DAI. **(B)** Comparison of sclerotium numbers among WT, Δ*Ssfkh1*, and Δ*Ssfkh1^com^*. Sclerotia were collected from 9-cm-diameter SPA plates for statistics analysis. Number of sclerotia in WT, Δ*Ssfkh1*, and Δ*Ssfkh1^com^* strains. **(C)** Comparison of sclerotium dry weights among WT, Δ*Ssfkh1*, and Δ*Ssfkh1^com^*. Sclerotia were collected from 9-cm-diameter SPA plates for statistics analysis. Number of sclerotia in WT, Δ*Ssfkh1*, and Δ*Ssfkh1^com^* strains. **(D)** Expression profiles of sclerotium-associated genes in SsFkh1 deletion mutants. Relative expression levels of sclerotium development-associated genes were investigated in hyphal tissue in Δ*Ssfkh1* mutant and WT strains. The experiments were performed in triplicate [^**^p < 0.01 (Student’s *t*-test)]. Values are means ± SE. **(E)** Pathogenicity phenotype of WT, Δ*Ssfkh1*, and Δ*Ssfkh1^com^* strains on cowpea and tomato leaves. Pictures were taken by 2 DAI when the lesions were observed under the agar plug. **(F)** Lesion diameters were measured on tomato leaves daily until 3 DAI. Values on the bars followed by the same letter are not significantly different at *p* = 0.05. The experiment was repeated three times. **(G)** Compound appressoria of WT, Δ*Ssfkh1*, and Δ*Ssfkh1^com^* strains on glass sides were observed by light microscopy at 1 DAI and compound appressorium development of WT, Δ*Ssfkh1*, and Δ*Ssfkh1^com^* strains on glass sides at 3 DAI using 5 mm diameter mycelial plugs.

To investigate the underlying mechanism involving in sclerotia development, which is mediated by SsFkh1, we generated the SsFkh1 deletion mutant Δ*Ssfkh1* and its complement strain Δ*Ssfkh1*^com^ by homologous recombination. Indeed, knockout of SsFkh1 led to impaired sclerotia development, as revealed by significantly reduced sclerotial numbers and dry weight, and reduced pathogenicity on its host cowpea plant. After 14 days, compared with WT, Δ*Ssfkh1* mutants produced less sclerotia, and only produced 60% dry weight of sclerotia as WT (Figures 1A–C). Moreover, in previous studies, some genes were reported involved in sclerotium development, including *Sssmk1* (Doehlemann et al., 2010), *Sspac1* (Rollins, 2003), *Ssrgb1* (Erental et al., 2007), *Sssac1* (Ii and Rollins, 2007), *Sspka1*, and *Sspka2* (Ii et al., 2004). Considering the defect of sclerotium development in Δ*Ssfkh1* mutant. We further profiled the expression of the various sclerotium development-associated genes mentioned above. The qRT-PCR results showed that the sclerotium-associated genes were significantly decreased expressed in Δ*Ssfkh1* mutant (Figure 1D). Larger lesions were caused in the WT and Δ*Ssfkh1*^com^ strains, but the Δ*Ssfkh1* mutant showed reduced virulence on cowpea and tomato leaves (Figures 1E,F). In addition, compared with WT, the number of compound appressorium was decreased, and not well developed (Figure 1G). These results indicated that the SsFkh1 may regulate the sclerotium development through sclerotium-associated genes, and affect compound appressorium development.

### Effect of Loss of SsFkh1 on Adapting to Stress in *Sclerotinia sclerotiorum*

**Figure 2:**
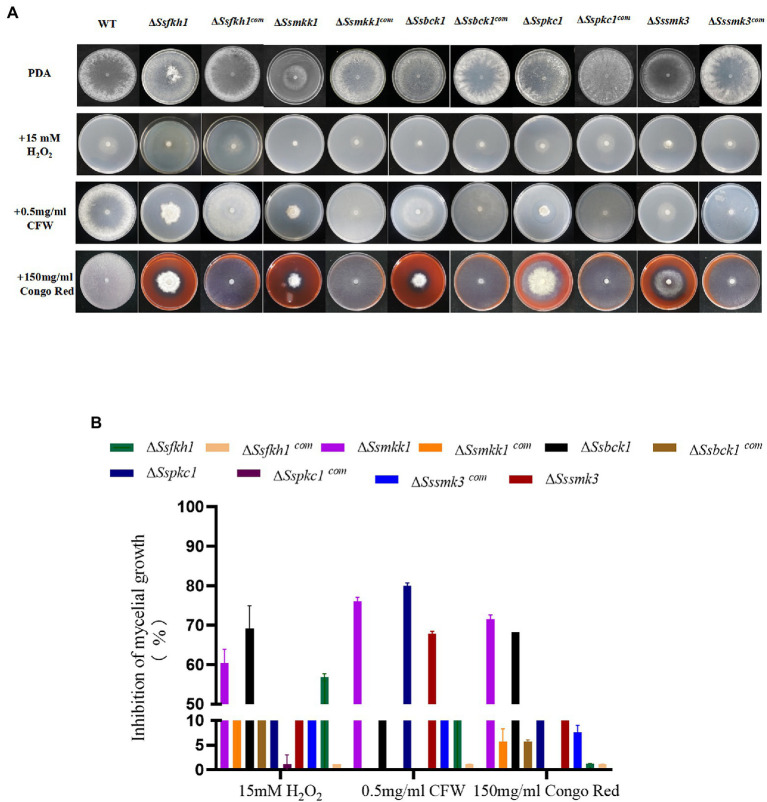
**(A)** Sensitivity of WT, Δ*Ssfkh1*, Δ*Ssmkk1*, Δ*Sspkc1*, Δ*Ssbck1*, Δ*Sssmk3*, Δ*Ssfkh1^com^*, Δ*Ssmkk1^com^*, Δ*Sspkc1^com^*, Δ*Ssbck1^com^*, and Δ*Sssmk3^com^* strains to 15 mM H_2_O_2_, 0.5 mg/ml Calcium fluoride white (CFW), 150 mg/ml Congo Red (CR) after incubation at 25°C for 3 days. Photographs were taken by 3 DAI. **(B)** Inhibition of mycelial growth of Δ*Ssfkh1*, Δ*Ssmkk1*, Δ*Sspkc1*, Δ*Ssbck1*, Δ*Sssmk3*, Δ*Ssfkh1^com^*, Δ*Ssmkk1^com^*, Δ*Sspkc1^com^*, Δ*Ssbck1^com^*, and Δ*Sssmk3^com^* strains. The experiment was repeated three times.

To evaluate the roles of SsFkh1 in adapting to stress, we compared the colony morphology of Δ*Ssfkh1* mutant, WT, and complemented strains on PDA with H_2_O_2_, Calcofluor white (CFW), and Congo red (CR). Compared with WT and complemented strains, Δ*Ssfkh1* mutant showed an obvious reduction in colony diameter under all stress conditions (Figures 2A,B). These results suggested that the SsFkh1 played an important role in cell wall integrity in *S. sclerotiorum*.

### Deletion of SsFkh1 Alters Gene Expression

**Figure 3:**
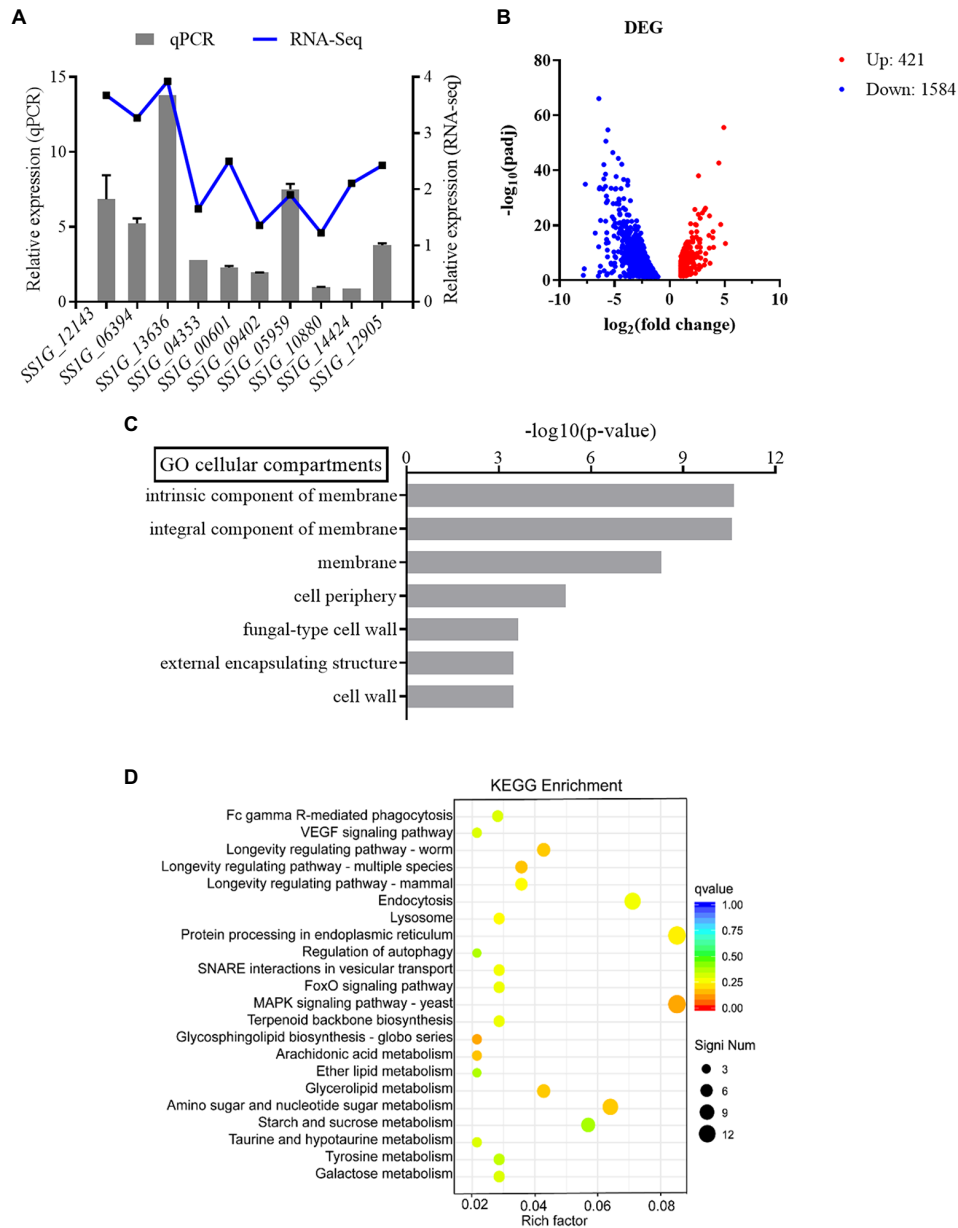
**(A)** The relative mRNA expression levels of DEGs and values of |Log2FC| (treatment group/control group) in RNA-seq. **(B)** Differential gene expression of WT and Δ*Ssfkh1*. Each point in the figure represents a gene, in which red indicates an upregulation gene, blue indicates a downregulation gene. **(C)** Gene ontology analysis (upregulation gene). Classification of the annotated amino acid sequences. Amino acid sequences were grouped into different functional subcategories: cellular component (CC), molecular function (MF), and biological process (BF). **(D)** KEGG pathway enrichment analysis.

In order to verify the reliability of transcriptome data, some genes were randomly selected from transcriptome data for verification. Several genes obtained from RNA-seq were selected for qRT-PCR analysis including SS1G_12143, SS1G_06394, SS1G_13636, SS1G_04353, SS1G_00601, SS1G_09402, SS1G_05959, SS1G_10880, SS1G_14424, and SS1G_12905. In general, the findings of qRT-PCR were consistent with those of transcriptome analysis, thereby demonstrating that RNA-Seq data was valid and accurate (Figure 3A). The RNA-Seq data were submitted to NCBI (accession # PRJNA795029).

Additionally, the differences in the gene expression patterns between the treatment and control groups were analyzed using read count data from the transcriptome. In total, 2,005 differentially expressed genes (DEGs) were assigned (value of *q* < 0.05, |FoldChange| > 2) in Δ*Ssfkh1* sample (Figure 3B). To analyze the functions of the DEGs, MF, CC, and BP were determined. Some membrane-related and cell wall-related components were enriched in the cellular component, indicating that Δ*Ssfkh1* strains may be involved in controlling the expression of cell wall-associated genes (Figure 3C). The results of the KEGG analysis revealed that “MAPK signaling pathway” was particularly enriched (Figure 3D). These results indicated SsFkh1 may be related to cell wall and MAPK pathway.

### SsFkh1 Interacts With SsMkk1

**Figure 4:**
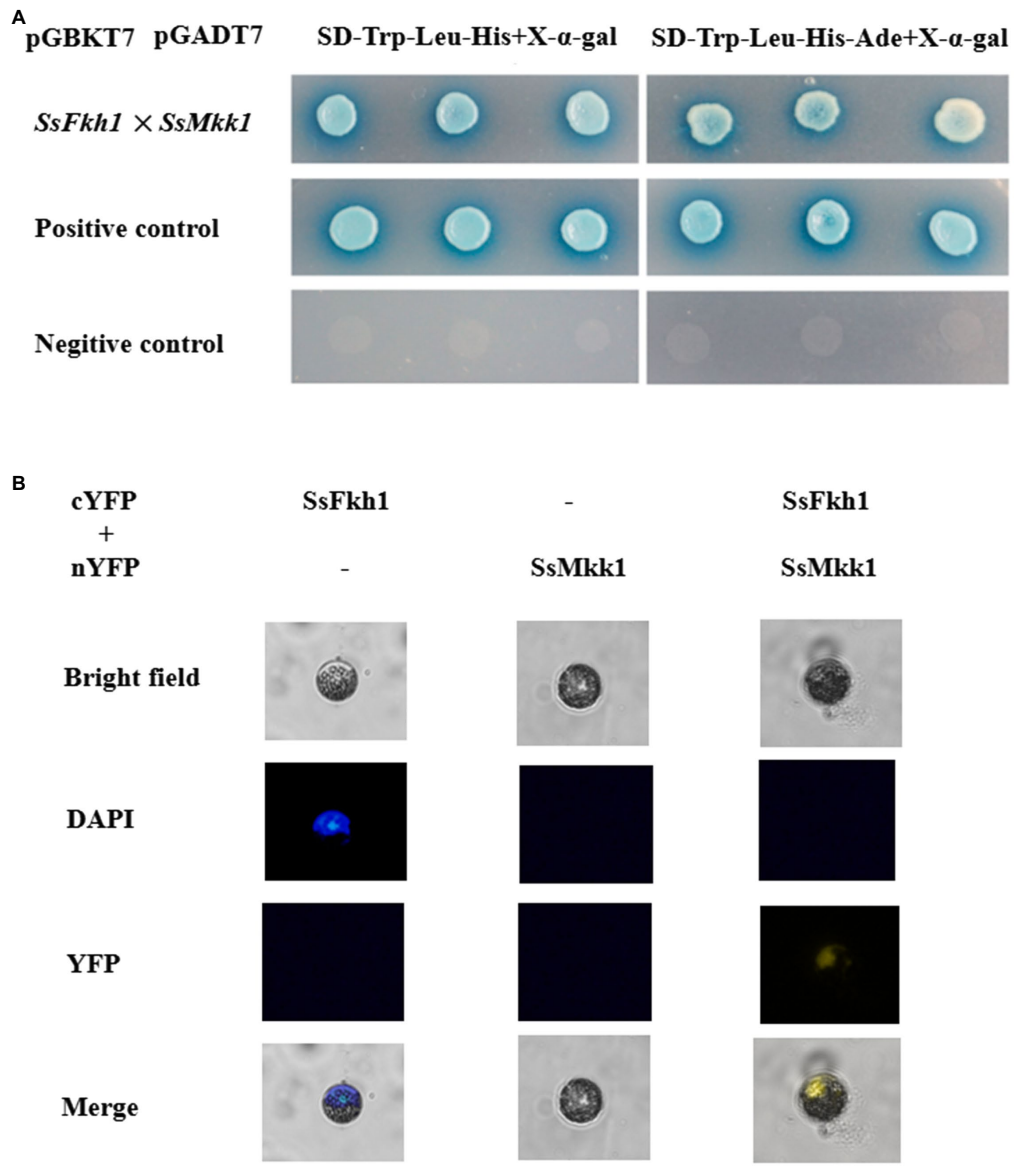
**(A)** Yeast two-hybrid (Y2H) analysis of interactions of SsFkh1 and SsMkk1. A pair of plasmids pGBKT7-53 and pGADT7 was used as a positive control. A pair of plasmids pGBKT7-Lam and pGADT7 was used as a negative control. **(B)** Confirmation of the interaction between SsFkh1 and SsMkk1 by bimolecular fluorescence complementation assay (BiFC) in *Arabidopsis* protoplast. Images were captured under a fluorescence microscope at 13 h after *Arabidopsis* protoplast transformation. The nucleus was visualized using DAPI (4=,6-diamidino-2-phenylindole) fluorescent dye. cYFP, C-terminal region of YFP; and nYFP, N-terminal region of YFP.

Cell wall integrity mediated by MAPK kinases is important not only for the integrity of cell wall, but also for the virulence, osmotic stress response, and development of some fungi. Based on the defects in cell wall integrity of Δ*Ssfkh1* mutant, we speculated SsFkh1 may interact with SsMkk1. The yeast two-hybrid assay was conducted to determine the interaction between SsFkh1 and SsMkk1. As shown in Figure 4, the SsFkh1 was interacted strongly with SsMkk1 in *S. sclerotiorum* (Figure 4A). In addition, a BiFC assay was used to further confirm the *in vivo* interaction between SsFkh1 and SsMkk1. Consistent with the result of yeast two-hybrid, BiFC also demonstrated the interaction between SsFkh1 and SsMkk1 (Figure 4B). Given the role of SsBck1 and SsSmk3 in MAPK signaling pathway, we also investigated the interaction between SsBck1 and SsSmk3 with SsFkh1; however, SsFkh1 exhibited no interaction with SsBck1 and SsSmk3 (data not shown).

### Deletion of SsMkk1, SsBck1, SsSmk3, and SsPkc1 Influence Sclerotium Development in *Sclerotinia sclerotiorum*

**Figure 5:**
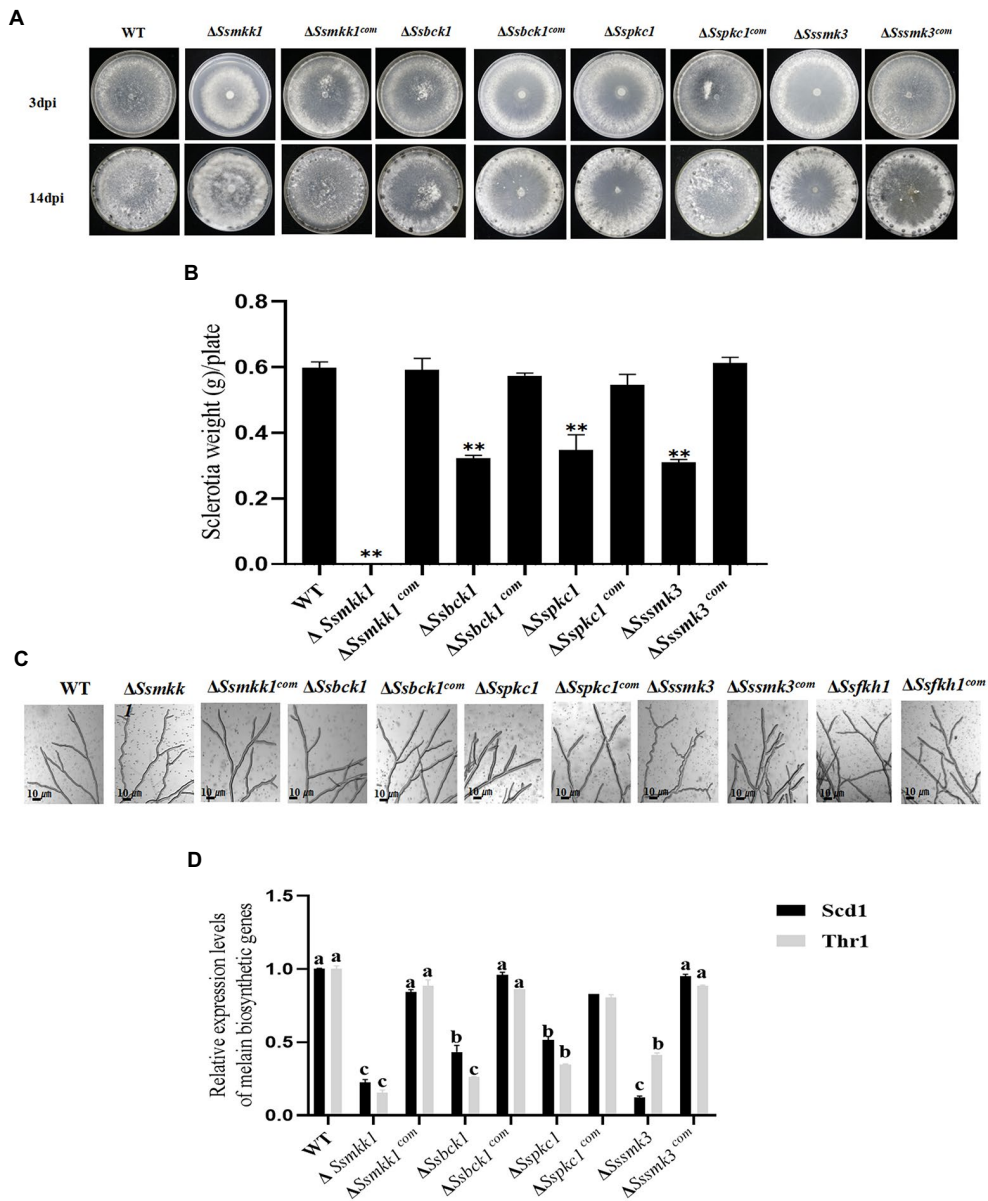
**(A)** Colony morphology of WT, Δ*Ssmkk1*, Δ*Sspkc1*, Δ*Ssbck1*, Δ*Sssmk3*, Δ*Ssmkk1^com^*, Δ*Sspkc1^com^*, Δ*Ssbck1^com^*, and Δ*Sssmk3^com^* on PDA after 3 and 14 days of incubation at 25°C. Photographs were taken by 3 DAI and 14 DAI. **(B)** Comparisons in sclerotia among the above strains on PDA plates after 14 days of incubation [** *p* < 0.01 (Student’s *t*-test)]. Values on the bars followed by the same letter are not significantly different at *p* = 0.05. **(C)** The observation of hyphae from WT, Δ*Ssmkk1*, Δ*Sspkc1*, Δ*Ssbck1*, Δ*Sssmk3*, Δ*Ssfkh1*, Δ*Ssmkk1^com^*, Δ*Sspkc1^com^*, Δ*Ssbck1^com^*, Δ*Ssfkh1^com^*, and Δ*Sssmk3^com^*. The photographs were taken after 16 h with a light microscope. Bar = 10 μm. **(D)** Comparisons of the transcript levels of two melanin biosynthesis-related genes *SCD1* and *THR1* among the above strains. The expression level of each gene in WT was referred to 1. Data are represented as mean values ± SE. Different letters indicate statistical significance (*p* < 0.05).

Based on the amino acid sequences of Pkc1, Bck1, Mkk1, and Smk3 from the budding yeast *S. cerevisiae*, SsMkk1 (SS1G_00059), SsBck1 (SS1G_10983), SsSmk3 (SS1G_05445), and SsPkc1 (SS1G_14026) were retrieved from the genome of *S. sclerotiorum*. SsMkk1, SsBck1, SsSmk3, and SsPkc1 are highly homologous to their counterparts from other fungal species (Supplementary Figure S1). To explore the function of SsMkk1, SsBck1, SsSmk3, and SsPkc1, the mutant strains were generated and verified by PCR (Supplementary Figure S2). In addition, to determine the function of SsMkk1, SsBck1, SsSmk3, and SsPkc1 in sclerotium development in *S. sclerotiorum*, the deletion mutants Δ*Ssmkk1*, Δ*Sspkc1*, Δ*Ssbck1*, Δ*Sssmk3*, and complemented strains were cultured on PDA plates. After 14 days, compared with WT, Δ*Sspkc1*, Δ*Ssbck1*, and Δ*Sssmk3* mutants produced less number of sclerotia, and only produced 58.2, 53.9, and 51.8% of dry weight of sclerotia as WT, respectively (Figures 5A,B). Even more, Δ*Ssmkk1* mutant completely lost the sclerotium-producing ability (Figures 5A,B). In addition, Δ*Ssmkk1* mutant showed a reduced growth rate on PDA and twisted hyphae morphology. Similar to Δ*Ssmkk1* mutant, Δ*Sssmk3* mutant also exhibited the twisted hyphae morphology, but no different growth rate (Figures 5A,C). These results indicated that SsMkk1 plays more essential roles in vegetative growth and sclerotia development than its upstream and downstream kinases SsBck1, SsPkc1, and SsSmk3, respectively.

Colony morphology assay exhibited that Δ*Ssmkk1*, Δ*Sspkc1*, Δ*Ssbck1*, and Δ*Sssmk3* produced less melanin in comparison with the wild type. This observation was further confirmed by determining the expression levels of two melanin biosynthesis-related genes *SCD1* and *THR1* in these mutants (Liang et al., 2017). Melanin accumulation is an important aspect of sclerotia formation because it increases the fungus’ resistance to harsh environmental conditions. qRT-PCR assays exhibited melanin biosynthetic gene downregulated expression in Δ*Ssmkk1*, Δ*Sspkc1*, Δ*Ssbck1*, and Δ*Sssmk3* mutants (Figure 5D), demonstrating that MAPKs SsMkk1, SsBck1, SsSmk3, and SsPkc1 regulate melanin biosynthesis in *S. sclerotiorum*.

### Δ*Ssmkk1* Exhibited Lower Virulence Than Δ*Sspkc1*, ΔSs*bck1*, and Δ*Sssmk3*

**Figure 6:**
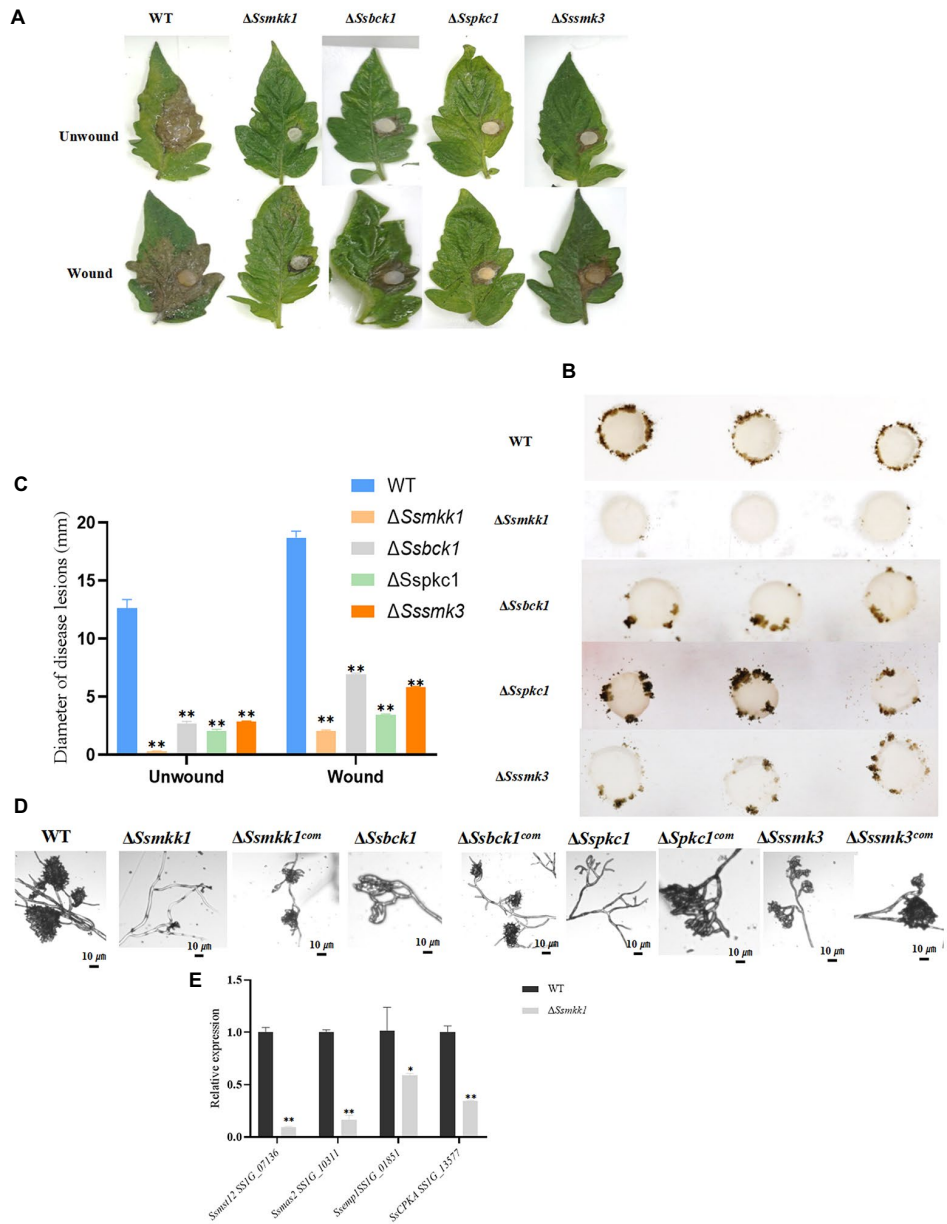
**(A)** WT, Δ*Ssmkk1*, Δ*Sspkc1*, Δ*Ssbck1*, and Δ*Sssmk3* strains were inoculated on unwounded and wounded common tomato leaves and photographed 2 DAI after inoculation. **(B)** Compound appressorium development of WT, Δ*Ssmkk1*, Δ*Sspkc1*, Δ*Ssbck1*, and Δ*Sssmk3* strains on glass sides. Compound appressoria were observed at 3 DAI using 5-mm-diameter mycelial plugs. **(C)** Diameters of disease lesions of WT, Δ*Ssmkk1*, Δ*Sspkc1*, Δ*Ssbck1*, and Δ*Sssmk3* strains. Values on the bars followed by the same letter are not significantly different at *p* = 0.05. The experiment was repeated three times. **(D)** Compound appressoria of WT, Δ*Ssmkk1*, Δ*Sspkc1*, Δ*Ssbck1*, Δ*Sssmk3*, Δ*Ssmkk1^com^*, Δ*Sspkc1^com^*, Δ*Ssbck1^com^*, and Δ*Sssmk3^com^* strains were observed on parafilm after 16 h. The photographs were taken after 16 hpi with a light microscope. Bar = 10 μm. **(E)** Relative expression levels of compound appressorium-associated genes were investigated in hyphal tissue in WT and Δ*Ssmkk1* strains. The constitutively expressed Actin was used as the reference gene to standardize data [^*^p < 0.05; ^**^p < 0.01 (Student’s *t*-test, *n* = 3)].

To verify the function of these four *S. sclerotiorum* MAPKs in pathogenicity, infection assay with wound and unwound tomato leaves was conducted. Detached tomato leaves were inoculated with mycelium plugs derived from WT, Δ*Ssmkk1*, Δ*Sspkc1*, Δ*Ssbck1*, and Δ*Sssmk3* mutants. Typical symptoms were caused in the WT strain, but the Δ*Ssmkk1*, Δ*Sspkc1*, Δ*Ssbck1*, and Δ*Sssmk3* mutants displayed reduced virulence on tomato leaves. Furthermore, the virulence of four mutants was rescued on wounded tomato leaves (Figures 6A,C).

Compound appressorium formed by hyphae play an important role in *S. sclerotiorum* penetration into the host. The WT and mutant strains were inoculated on glass slide to induce compound appressorium. Compared with WT, the Δ*Ssmkk1* mutant failed to produce compound appressorium, and Δ*Sspkc1*, Δ*Ssbck1*, and Δ*Sssmk3* mutants produced less mature compound appressorium (Figures 6B,D). These results indicated that SsMkk1, SsBck1, SsSmk3, and SsPkc1 are necessary for compound appressorium development and virulence in *S. sclerotiorum*. Furthermore, the expression of genes involved in compound appressorium formation was analyzed in WT and Δ*Ssmkk1* mutant, including *Ssmst12*, *Ssmas2*, *Ssemp1*, and *Sscpka*. The expression level of all these four genes were dramatically decreased in Δ*Ssmkk1* mutant. These results provided a strong support to the defect in compound appressorium development of Δ*Ssmkk1* mutant (Figure 6E).

### Disruption of SsMkk1, SsBck1, SsSmk3, and SsPkc1 Leads to Defective Cell Wall Integrity

To determine the role of SsMkk1, SsBck1, SsSmk3, and SsPkc1in the maintenance of the cell wall integrity in *S. sclerotiorum*, the sensitivity of Δ*Ssmkk1*, Δ*Sspkc1*, Δ*Ssbck1*, and Δ*Sssmk3* to cell wall-damaging compounds was analyzed. Compared with WT, all the four mutants were more sensitive to CFW and CR. In addition, the Δ*Ssmkk1* mutant showed obvious reduction in colony diameter under H_2_O_2_ stress. Δ*Ssbck1* was also more sensitive to H_2_O_2_ stress (Figures 2A,B). The morphology and growth of complemented strains was restored to WT levels on all the stress media.

## Discussion

We characterized the functions of SsFkh1 in *S. sclerotiorum* in the context of sclerotium development and pathogenicity. Moreover, CWI pathway mediated by MAPK kinases was enriched in transcriptome analysis. The conserved CWI pathway is responsible for the integrity of cell wall in many fungal species. Similar to the reports from other fungi, the MAPK mutants in the CWI pathway of *S. sclerotiorum* showed increased sensitivity to CR and CFW (Yun et al., 2013; Zhang et al., 2017; Valiante et al., 2020). Previous studies have shown that the three MAPKs Bck1, Mkk1, and Slt2 function clearly in the same way in *S. cerevisiae*, *Aspergillus fumigatus*, *Magnaporthe oryzae*, *Ustilago maydis*, and *Ashbya gossypii*. Moreover, the three MAPK mutants of *Fusarium graminearum* or *M. oryzae* showed similar morphological characters. MAPK kinase mutant (BcMkk1) exhibits more severe defects in mycelial growth, conidiation, responses to cell wall, and oxidative stresses, but has less reduced virulence than mutants of its upstream (BcBck1) and downstream (BcBmp3) kinases in *Botrytis cinerea* (Yin et al., 2018). In this study, when compared to the WT and complemented strains, the number of sclerotia in the Δ*Sspkc1*, Δ*Ssbck1*, and Δ*Sssmk3* mutants decreased significantly (Figure 5A). Interestingly, Δ*Ssmkk1* did not produce sclerotia and produced a very small number of compound appressorium. Different from BcMkk1 of *B. cinerea*, Δ*Ssmkk1* showed lower virulence than Δ*Sspkc1*, Δ*Ssbck1*, and Δ*Sssmk3* mutants (Figure 6A). Moreover, Δ*Ssmkk1* was more sensitive to cell wall stimulation (Figure 2). These evidences suggested that SsMkk1 may be an important kinase in MAPK pathway. In addition, transcriptome analysis showed that knockout of SsFkh1 resulted in a large enrichment of CWI pathway mediated by MAPK kinases. And yeast two-hybrid and BiFC assay confirmed the interaction between SsMkk1 and SsFkh1 (Figure 4). Therefore, we investigated the relationship between SsFkh1 and related proteins involved in MAPK pathway. However, the role of SsFkh1 which played in CWI pathway mediated by MAPK kinases remains to be further studied.

Sclerotium development include six stages and is tightly regulated by many genetic factors (Li and Rollins, 2009). Loss of the ability to produce normal sclerotia in *S. sclerotiorum* may disrupt the disease cycle and the ability to cause disease (Rollins, 2003; Erental et al., 2007; Li et al., 2018). In this study, we found that the Δ*Ssmkk1* mutant lost its ability to produce sclerotia and infection cushion and exhibited severely impaired pathogenicity compared to the WT and complemented strains (Figure 5). Similarly, after deletion of its homologous protein, MoMkk1, *M. oryzae* could produce less aerial hyphal, cause defective asexual development, and attenuated pathogenicity (Yang et al., 2018). Moreover, some sclerotium development-associated genes were reported. For example, the ERK like MAPK gene *Smk1* was obtained from *S. sclerotiorum*, which showed that *Smk1* was necessary for sclerotium development, and further clarified that the regulation of *Smk1* on sclerotium development was through the pH-dependent signal pathway of OA accumulation (Chen et al., 2004). Previous studies have shown that sclerotium development was influenced by various environmental changes, primary metabolites, and secondary metabolites in *S. sclerotiorum* (Erental et al., 2008). The inability of *S. sclerotiorum* to form normal sclerotia might affect the disease cycle and the potential to cause illness (Zhu et al., 2019). OA plays a critical role in the infection process of *S. sclerotiorum* (Fagundes-Nacarath et al., 2018; Liang and Rollins, 2018). The Ssnox1 mutant has been reported to display lower oxalate production and reduced pathogenicity (Hyo-jin et al., 2011). Moreover, the knockout mutant of adenylate cyclase gene *Sac1* seriously affected mycelial growth, sclerotia development, and pathogenicity in *S. sclerotiorum* (Rollins and Dickman, 1998). In this study, Δ*Ssmkk1* strains produced less OA than other knockout mutants and WT (data not shown). However, this may be related to the slow growth of Δ*Ssmkk1* hypha, and the amount of OA could be determined in the further experiment. In addition, melanin protects mycelia and sclerotia from some adverse environmental conditions. The genes involved in melanin biosynthesis, *SCD1* encoding a scytalone dehydratase and *THR1* encoding a trihydroxynaphthalene reductase, disruption of *SCD1* or *THR1* impaired sclerotial development in *S. sclerotiorum* (Liang et al., 2017). Melanin is a necessary condition for sclerotia formation. The qRT-PCR results showed that the sclerotium-associated genes were significantly decreased expressed in Δ*Ssmkk1*, Δ*Sspkc1*, Δ*Ssbck1*, and Δ*Sssmk3* mutant strains (Figure 5D). MAPK pathway may affect sclerotia formation by regulating the expression of melanin synthesis genes. Δ*Ssfkh1* also showed sclerotia defects, and the downregulation of sclerotia related genes.

Compound appressoria are hyphal tip-differentiated multicellular infection structures formed by numerous plant pathogenic fungi on the host surface (Hofman and Jongebloed, 1988; Boenisch and Schäfer, 2011). Impenetrable surfaces, such as dialysis tubing, parafilm, plastics, glass, and cellophane, are frequently effective in triggering compound appressoria differentiation in *S. sclerotiorum* (Liu et al., 2018). Compound appressoria is essential for penetrating host cells, and the complexity of compound appressoria is related to physical resistance to penetration on the host surface (Tariq and Jeffries, 1984; Jamaux et al., 1995; Huang et al., 2016). Some genes affect the formation and development of compound appressoria. The glutathione transpeptidase gene *SsGgt1* of *S. sclerotiorum* is necessary for the accumulation of glutathione, the development of sclerotia, and the formation of compound appressoria. There was no compound appressoria formation after knockout of the gene (Moyi and Rollins, 2010). The compound appressorium formation-related gene 1, *Sscaf1*, which encodes a secretory protein and involved in to infection cushions formation, plays a crucial role in host penetration (Xiao et al., 2014). In this study, Δ*Ssfkh1*, Δ*Ssmkk1*, Δ*Sspkc1*, Δ*Ssbck1*, and Δ*Sssmk3* mutant strains produce less compound appressorium compared with WT and complement mutants (Figures 1G, 6B). In addition, the virulence of the Δ*Ssfkh1*, Δ*Ssmkk1*, Δ*Sspkc1*, Δ*Ssbck1*, and Δ*Sssmk3* mutant strains were weakened (Figures 1E, 6A).

In conclusion, Δ*Ssmkk1*, Δ*Sspkc1*, Δ*Ssbck1*, and Δ*Sssmk3* maintained the phenotype to produce a small number of sclerotia and exhibited decreased pathogenicity. These phenotypes are consistent with Δ*Ssfkh1*. Moreover, transcriptome analysis showed that deletion of SsFkh1 resulted in a large enrichment of CWI pathway mediated by MAPK kinases. Therefore, we explored the MAPK pathway and discovered that SsFkh1 was directly interacting with SsMkk1. Furthermore, the SsBck1 and SsPkc1 phenotypes showed reduced MAPK pathway activity upstream of SsMkk1, which may indicate that there might be several MAPKKKs acting upstream of SsMkk1, although SsMkk1 appears to be the main kinase. On the one hand, SsFkh1 can control the expression of certain genes and, on the other, it may also preserve cell wall integrity. And we will get more evidence to confirm the relationship between SsFkh1 and the CWI pathway mediated by MAPK kinases in the further experiments. In a word, SsFkh1 may be participate in the CWI pathway mediated by MAPK kinases. This provides an important basis for analyzing the development mechanism of sclerotia and compound appressorium of *S. sclerotiorum* in the future.

## Data Availability Statement

The original contributions presented in the study are included in the article/**Supplementary Material**, further inquiries can be directed to the corresponding author.

## Author Contributions

JC and HP planned and designed the research and wrote the manuscript. XZ modified the manuscript. All authors performed the experiments and analyzed the data. All authors contributed to the article and approved the submitted version.

## Funding

This work was financially supported by National Natural Science Foundation of China (31772108, 31972978), grants from Inter-governmental International Cooperation Special Project of National Key R & D Program of China (2019YFE0114200), the Thirteenth National Key R & D Program of China (2019YFD1002000), and Science and Technology Development Plan of Jilin Province (20190201025JC).

## Conflict of Interest

The authors declare that the research was conducted in the absence of any commercial or financial relationships that could be construed as a potential conflict of interest.

## Publisher’s Note

All claims expressed in this article are solely those of the authors and do not necessarily represent those of their affiliated organizations, or those of the publisher, the editors and the reviewers. Any product that may be evaluated in this article, or claim that may be made by its manufacturer, is not guaranteed or endorsed by the publisher.

## Footnotes

^1^ http://www.geneontology.org/

^2^ http://www.genome.jp/kegg/

## Supplementary Material

The Supplementary Material for this article can be found online at: https://www.frontiersin.org/articles/10.3389/fmicb. 2022.816091/full#supplementary-material

## References

[ref2] Arsenault H. E. Roy J. Mapa C. E. Cyert M. S. Benanti J. A. (2015). Hcm1 integrates signals from Cdk1 and calcineurin to control cell proliferation. Mol. Biol. Cell 26, 3570–3577. doi: 10.1091/mbc.E15-07-0469, PMID: 26269584 PMC4603928

[ref3] Bensen E. S. Filler S. G. Berman J. (2002). A forkhead transcription factor is important for true hyphal as well as yeast morphogenesis in *Candida albicans*. Eukaryot. Cell 1, 787–798. doi: 10.1128/EC.1.5.787-798.2002, PMID: 12455696 PMC126749

[ref4] Boenisch M. J. Schäfer W. (2011). *Fusarium graminearum* forms mycotoxin producing infection structures on wheat. BMC Plant Biol. 11:110. doi: 10.1186/1471-2229-11-110, PMID: 21798058 PMC3166921

[ref5] Bolton M. Thomma B. Nelson B. D. (2010). *Sclerotinia sclerotiorum* (Lib.) de Bary: biology and molecular traits of a cosmopolitan pathogen. Mol. Plant Pathol. 7, 1–16. doi: 10.1111/j.1364-3703.2005.00316.x, PMID: 20507424

[ref6] Carlsson P. Mahlapuu M. (2002). Forkhead transcription factors: key players in development and metabolism. Dev. Biol. 250, 1–23. doi: 10.1006/dbio.2002.0780, PMID: 12297093

[ref8] Chen C. Harel A. Gorovoits R. Yarden O. Dickman M. B. (2004). MAPK regulation of sclerotial development in *Sclerotinia sclerotiorum* is linked with pH and cAMP sensing. Mol. Plant-Microbe Interact. 17, 404–413. doi: 10.1094/MPMI.2004.17.4.404, PMID: 15077673

[ref9] Doehlemann G. Berndt P. Hahn M. (2010). Different signalling pathways involving a Gα protein, cAMP and a MAP kinase control germination of *Botrytis cinerea* conidia. Mol. Microbiol. 59, 821–835. doi: 10.1111/j.1365-2958.2005.04991.x 16420354

[ref10] Erental A. Dickman M. B. Yarden O. (2008). Sclerotial development in *Sclerotinia sclerotiorum*: awakening molecular analysis of a "dormant" structure. Fungal Biol. Rev. 22, 6–16. doi: 10.1016/j.fbr.2007.10.001

[ref11] Erental A. Harel A. Yarden O. (2007). Type 2A phosphoprotein phosphatase is required for asexual development and pathogenesis of *Sclerotinia sclerotiorum*. Mol. Plant-Microbe Interact. 20, 944–954. doi: 10.1094/MPMI-20-8-0944, PMID: 17722698

[ref100] Fan H. Yu G. Liu Y. Zhang X. Liu J. Zhang Y.. (2017). An atypical forkhead-containing transcription factor SsFKH1 is involved in sclerotial formation and is essential for pathogenicity in Sclerotinia sclerotiorum. Mol. Plant Pathol. 18, 963–975. doi: 10.1111/mpp.12453, PMID: 27353472 PMC6638265

[ref12] Fagundes-Nacarath I. R. F. Debona D. Rodrigues F. A. (2018). Oxalic acid-mediated biochemical and physiological changes in the common bean- *Sclerotinia sclerotiorum* interaction. Plant Physiol. Biochem. 129, 109–121. doi: 10.1016/j.plaphy.2018.05.028, PMID: 29870862

[ref13] Fan J. Y. Cui Z. Q. Wei H. P. Zhang Z. P. Zhang X. E. (2008). Split mCherry as a new red bimolecular fluorescence complementation system for visualizing protein-protein interactions in living cells. Biochem. Biophys. Res. Commun. 367, 47–53. doi: 10.1016/j.bbrc.2007.12.101, PMID: 18158915

[ref15] Fuchs B. B. Mylonakis E. (2009). Our paths might cross: the role of the fungal cell wall integrity pathway in stress response and cross talk with other stress response pathways. Eukaryot. Cell 8, 1616–1625. doi: 10.1128/EC.00193-09, PMID: 19717745 PMC2772411

[ref17] Gu Q. Chen Y. Liu Y. Zhang C. Ma Z. (2015). The transmembrane protein FgSho1 regulates fungal development and pathogenicity via the MAPK module Ste50-Ste11-Ste7 in *Fusarium graminearum*. New Phytol. 206, 315–328. doi: 10.1111/nph.13158, PMID: 25388878

[ref18] Guogen Y. Liguang T. Yingdi G. Xie J. Fu Y. Jiang D.. (2017). A cerato-platanin protein SsCP1 targets plant PR1 and contributes to virulence of *Sclerotinia sclerotiorum*. New Phytol. 217, 739–755. doi: 10.1111/nph.14842, PMID: 29076546

[ref19] Guyon K. Balague C. Roby D. Raffaele S. (2014). Secretome analysis reveals effector candidates associated with broad host range necrotrophy in the fungal plant pathogen *Sclerotinia sclerotiorum*. BMC Genomics 15:336. doi: 10.1186/1471-2164-15-336, PMID: 24886033 PMC4039746

[ref20] Hisano Y. Sakuma T. Nakade S. Ohga R. Ota S. Okamoto H.. (2015). Precise in-frame integration of exogenous DNA mediated by CRISPR/Cas9 system in zebrafish. Sci. Rep. 5:8841. doi: 10.1038/srep08841, PMID: 25740433 PMC4350073

[ref21] Hofman T. W. Jongebloed P. (1988). Infection process of *Rhizoctonia solani* on *Solanum tuberosum* and effects of granular nematicides. Neth. J. Plant Pathol. 94, 243–252. doi: 10.1007/BF01977314

[ref22] Hou J. Feng H. Q. Chang H. W. Liu Y. Li G. H. Yang S.. (2020). The H3K4 demethylase Jar1 orchestrates ROS production and expression of pathogenesis-related genes to facilitate *Botrytis cinerea* virulence. New Phytol. 225, 930–947. doi: 10.1111/nph.16200, PMID: 31529514

[ref23] Huang L. Buchenauer H. Han Q. Zhang X. Kang Z. (2016). Ultrastructural and cytochemical studies on the infection process of *Sclerotinia sclerotiorum* in oilseed rape. J. Plant Dis. Protect. 115, 9–16. doi: 10.1007/BF03356233

[ref25] Hyo-jin K. Changbin C. Mehdi K. Dickman M. B. (2011). Identification and characterization of *Sclerotinia sclerotiorum* NADPH oxidases. Appl. Environ. Microbiol. 77, 7721–7729. doi: 10.1128/AEM.05472-11, PMID: 21890677 PMC3209176

[ref27] Ii W. M. J. Dickman M. B. Rollins J. A. (2004). Characterization and functional analysis of a cAMP-dependent protein kinase A catalytic subunit gene (*pka1*) in *Sclerotinia sclerotiorum*. Physiol. Mol. Plant Pathol. 64, 155–163. doi: 10.1016/j.pmpp.2004.07.004

[ref28] Ii W. M. J. Rollins J. A. (2007). Deletion of the adenylate cyclase (*sac1*) gene affects multiple developmental pathways and pathogenicity in *Sclerotinia sclerotiorum*. Fungal Genet. Biol. 44, 521–530. doi: 10.1016/j.fgb.2006.11.005, PMID: 17178247

[ref29] Jamaux I. Gelie B. Lamarque C. (1995). Early stages of infection of rapeseed petals and leaves by *Sclerotinia sclerotiorum* revealed by scanning electron microscopy. Plant Pathol. 44, 22–30. doi: 10.1111/j.1365-3059.1995.tb02712.x

[ref30] Jendretzki A. Wittland J. Wilk S. Straede A. Heinisch J. J. (2011). How do I begin? Sensing extracellular stress to maintain yeast cell wall integrity. Eur. J. Cell Biol. 90, 740–744. doi: 10.1016/j.ejcb.2011.04.006, PMID: 21640429

[ref31] Jeon J. Goh J. Yoo S. Chi M. H. Choi J. Rho H. S.. (2008). A putative MAP kinase kinase kinase, MCK1, is required for cell wall integrity and pathogenicity of the rice blast fungus, *Magnaporthe oryzae*. Mol. Plant-Microbe Interact. 21, 525–534. doi: 10.1094/MPMI-21-5-0525, PMID: 18393612

[ref35] Levin D. E. (2005). Cell wall integrity signaling in *Saccharomyces cerevisiae*. Microbiol. Mol. Biol. Rev. 69, 262–291. doi: 10.1128/MMBR.69.2.262-291.2005, PMID: 15944456 PMC1197416

[ref36] Levin D. E. (2011). Regulation of cell wall biogenesis in *Saccharomyces cerevisiae*: the cell wall integrity signaling pathway. Genetics 189, 1145–1175. doi: 10.1534/genetics.111.128264, PMID: 22174182 PMC3241422

[ref37] Li J. Mu W. Veluchamy S. Liu Y. Zhang Y. Pan H.. (2018). The GATA-type IVb zinc-finger transcription factor SsNsd1 regulates asexual-sexual development and appressoria formation in *Sclerotinia sclerotiorum*. Mol. Plant Pathol. 19, 1679–1689. doi: 10.1111/mpp.12651, PMID: 29227022 PMC6638148

[ref38] Li M. Rollins J. A. (2009). The development-specific protein (Ssp1) from *Sclerotinia sclerotiorum* is encoded by a novel gene expressed exclusively in sclerotium tissues. Mycologia 101, 34–43. doi: 10.3852/08-114, PMID: 19271669

[ref40] Liang X. Rollins J. A. (2018). Mechanisms of broad host range necrotrophic pathogenesis in *Sclerotinia sclerotiorum*. Phytopathology 108, 1128–1140. doi: 10.1094/PHYTO-06-18-0197-RVW, PMID: 30048598

[ref41] Liang Y. Xiong W. Steinkellner S. Feng J. (2017). Deficiency of the melanin biosynthesis genes *SCD1* and *THR1* affects sclerotial development and vegetative growth, but not pathogenicity, in *Sclerotinia sclerotiorum*. Mol. Plant Pathol. 19, 1444–1453. doi: 10.1111/mpp.12627, PMID: 29024255 PMC6638068

[ref42] Liu L. Wang Q. Zhang X. Liu J. Zhang Y. Pan H. (2018). *Ssams2*, a gene encoding GATA transcription factor, is required for appressoria formation and chromosome segregation in *Sclerotinia sclerotiorum*. Front. Microbiol. 9:3031. doi: 10.3389/fmicb.2018.03031, PMID: 30574138 PMC6291475

[ref200] Liu Y. G. Chen Y. (2007). High-efficiency thermal asymmetric interlaced PCR for amplification of unknown flanking sequences. Biotechniques 43: 649., PMID: 18072594 10.2144/000112601

[ref45] Moyi L. Rollins J. A. (2010). The development-specific *ssp1* and *ssp2* genes of *Sclerotinia sclerotiorum* encode lectins with distinct yet compensatory regulation. Fungal Genet. Biol. 47, 531–538. doi: 10.1016/j.fgb.2010.03.008, PMID: 20350614

[ref46] Park J. Kong S. Kim S. Kang S. Lee Y. H. (2014). Roles of forkhead-box transcription factors in controlling development, pathogenicity, and stress response in *Magnaporthe oryzae*. Plant Pathol. J. 30, 136–150. doi: 10.5423/PPJ.OA.02.2014.0018, PMID: 25288996 PMC4174854

[ref47] Pataki E. Miklos I. Weisman R. Sipiczki M. (2017). fhl1 gene of the fission yeast regulates transcription of meiotic genes and nitrogen starvation response, downstream of the TORC1 pathway. Curr. Genet. 63, 91–101. doi: 10.1007/s00294-016-0607-1, PMID: 27165118

[ref48] Postnikoff S. Malo M. E. Wong B. Harkness T. Copenhaver G. P. (2012). The yeast forkhead transcription factors Fkh1 and Fkh2 regulate lifespan and stress response together with the anaphase-promoting complex. PLoS Genet. 8:e1002583. doi: 10.1371/journal.pgen.1002583, PMID: 22438832 PMC3305399

[ref50] Rollins J. A. (2003). The *Sclerotinia sclerotiorum pac1* gene is required for sclerotial development and virulence. Mol. Plant-Microbe Interact. 16, 785–795. doi: 10.1094/MPMI.2003.16.9.785, PMID: 12971602

[ref51] Rollins J. A. Dickman M. B. (1998). Increase in endogenous and exogenous cyclic AMP levels inhibits sclerotial development in *Sclerotinia sclerotiorum*. Appl. Environ. Microbiol. 64, 2539–2544. doi: 10.1128/AEM.64.7.2539-2544.1998, PMID: 9647827 PMC106423

[ref53] Sanz A. B. García R. Rodríguez-Pea J. M. Nombela C. Arroyo J. (2016). Cooperation between SAGA and SWI/SNF complexes is required for efficient transcriptional responses regulated by the yeast MAPK Slt2. Nucleic Acids Res. 44, 7159–7172. doi: 10.1093/nar/gkw324, PMID: 27112564 PMC5009723

[ref54] Sanz A. B. Garcia R. Rodriguez-Pena J. M. Diez-Muniz S. Nombela C. Peterson C. L.. (2012). Chromatin remodeling by the SWI/SNF complex is essential for transcription mediated by the yeast cell wall integrity MAPK pathway. Mol. Biol. Cell 23, 2805–2817. doi: 10.1091/mbc.e12-04-0278, PMID: 22621902 PMC3395667

[ref55] Smith M. E. Henkel T. W. Rollins J. A. (2015). How many fungi make sclerotia? Fungal Ecol. 13, 211–220. doi: 10.1016/j.funeco.2014.08.010, PMID: 35134302

[ref57] Takatani T. Takahashi K. Uozumi Y. Matsuda T. Ito T. Schaffer S. W.. (2004). Taurine prevents the ischemia-induced apoptosis in cultured neonatal rat cardiomyocytes through Akt/caspase-9 pathway. Biochem. Biophys. Res. Commun. 316, 484–489. doi: 10.1016/j.bbrc.2004.02.066, PMID: 15020243

[ref58] Tariq V. N. Jeffries P. (1984). Appressorium formation by *Sclerotinia sclerotiorum*: scanning electron microscopy. Trans. Br. Mycol. Soc. 82, 645–651. doi: 10.1016/S0007-1536(84)80105-9

[ref61] Valiante V. Macheleidt J. Föge M. Brakhage A. A. (2020). The *Aspergillus fumigatus* cell wall integrity signaling pathway: drug target, compensatory pathways, and virulence. Front. Microbiol. 6:325. doi: 10.3389/fmicb.2015.00325, PMID: 25932027 PMC4399325

[ref62] Wang L. Liu Y. Liu J. Zhang Y. Pan H. (2016). The *Sclerotinia sclerotiorum FoxE2* gene is required for apothecial development. Phytopathology 106, 484–490. doi: 10.1094/PHYTO-08-15-0181-R, PMID: 26756829

[ref65] Xiao X. Xie J. Cheng J. Li G. Fu Y. (2014). Novel secretory protein Ss-Caf1 of the plant-pathogenic fungus *Sclerotinia sclerotiorum* is required for host penetration and normal sclerotial development. Mol. Plant-Microbe Interact. 27, 40–55. doi: 10.1094/MPMI-05-13-0145-R, PMID: 24299212

[ref66] Yang J. Liu M. Liu X. Yin Z. Sun Y. Zhang H.. (2018). Heat-shock proteins MoSsb1, MoSsz1, and MoZuo1 attenuate MoMkk1-mediated cell-wall integrity signaling and are important for growth and pathogenicity of *Magnaporthe oryzae*. Mol. Plant-Microbe Interact. 31, 1211–1221. doi: 10.1094/MPMI-02-18-0052-R, PMID: 29869941 PMC6790631

[ref67] Yin Y. Wu S. Chaonan C. Ma T. Jiang H. Matthias H.. (2018). The MAPK kinase BcMkk1 suppresses oxalic acid biosynthesis via impeding phosphorylation of BcRim15 by BcSch9 in *Botrytis cinerea*. PLoS Pathog. 14:e1007285. doi: 10.1371/journal.ppat.1007285, PMID: 30212570 PMC6136818

[ref68] Yoo S. D. Cho Y. H. Sheen J. (2007). *Arabidopsis* mesophyll protoplasts. Nat. Protoc. 2, 1565–1572. doi: 10.1038/nprot.2007.199, PMID: 17585298

[ref69] Yun Y. Liu Z. Zhang J. Shim W. B. Ma Z. (2013). The MAPKK FgMkk1 of *Fusarium graminearum* regulates vegetative differentiation, multiple stress response, and virulence via the cell wall integrity and high-osmolarity glycerol signaling pathways. Environ. Microbiol. 16, 2023–2037. doi: 10.1111/1462-2920.12334, PMID: 24237706

[ref70] Zhang H. Li Y. Lai W. Huang K. Li Y. Wang Z.. (2021). SsATG8 and SsNBR1 mediated-autophagy is required for fungal development, proteasomal stress response and virulence in *Sclerotinia sclerotiorum*. Fungal Genet. Biol. 157:103632. doi: 10.1016/j.fgb.2021.103632, PMID: 34710583

[ref71] Zhang G. Sun Z. Ren A. Shi L. Shi D. Li X.. (2017). The mitogen-activated protein kinase GlSlt2 regulates fungal growth, fruiting body development, cell wall integrity, oxidative stress and ganoderic acid biosynthesis in *Ganoderma lucidum*. Fungal Genet. Biol. 104, 6–15. doi: 10.1016/j.fgb.2017.04.004, PMID: 28435030

[ref72] Zhu W. Wei W. Fu Y. Cheng J. Xie J. Li G.. (2013). A secretory protein of necrotrophic fungus *Sclerotinia sclerotiorum* that suppresses host resistance. PLoS One 8:e53901. doi: 10.1371/journal.pone.0053901, PMID: 23342034 PMC3544710

[ref73] Zhu G. Yu G. Zhang X. Liu J. Zhang Y. Rollins J. A.. (2019). The formaldehyde dehydrogenase SsFdh1 is regulated by and functionally cooperates with the GATA transcription factor SsNsd1 in *Sclerotinia sclerotiorum*. mSystems 4, e00397–e00419. doi: 10.1128/mSystems.00397-19, PMID: 31506263 PMC6739101

