# Supplementary material for: The Coupling Between Cell Wall Integrity Mediated by MAPK Kinases and SsFkh1 Is Involved in Sclerotia Formation and Pathogenicity of Sclerotinia sclerotiorum
Source: Front Microbiol. 2022 Apr 25;13:816091. doi: 10.3389/fmicb.2022.816091 (PMC9081980; doi:10.3389/fmicb.2022.816091)
Supplement: Supplementary file 1 [file Data_Sheet_1.docx]

Supplementary Material

**
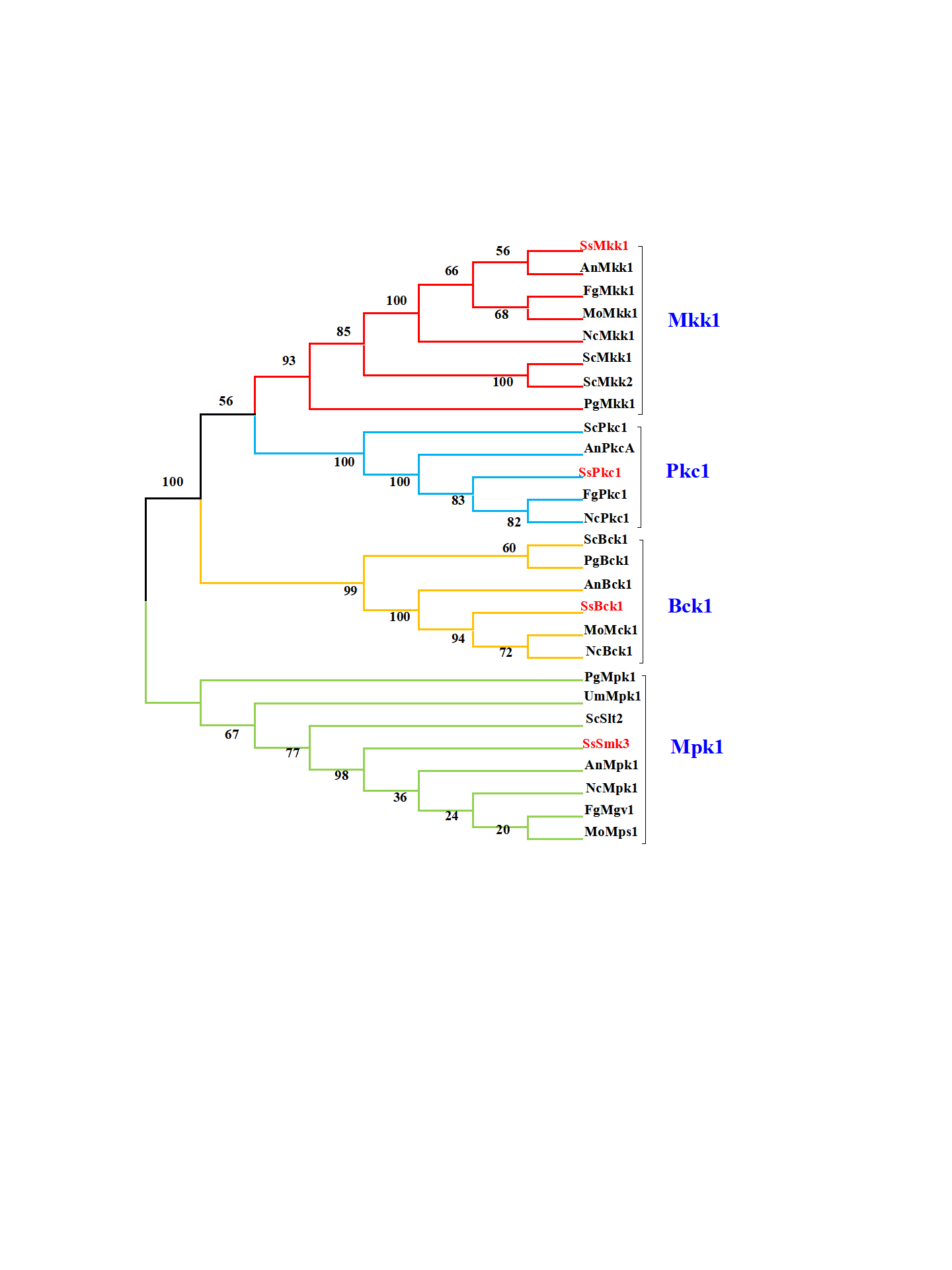
**

**Supplementary Figure 1.** *S. sclerotiorum* mitogen-activated protein kinases (MAPKs) in the cell wall integrity (CWI) pathway are homologous to those counterparts from yeasts and other filamentous fungi. Phylogenetic tree generated using the neighbor-joining method with Mega-X software on the basis of the deduced amino acid sequences of MAPK orthologs from different fungi. SsBck1(GenBank accession no. XP_001587743.1), SsMkk1 (XP_001597973.1), SsSmk3 (XP_001594017.1) and SsPkc1 (XP_001584929.1) from *S. sclerotiorum* that were indicated with red font; ScBck1 (NP_012440.1), ScPkc1 (NP_009445.2), ScMkk1 (NP_014874.1), ScMkk2 (NP_015185.1) and ScSlt2 (NP_011895.1) from *Saccharomyces cerevisiae*; MoMck1 (ELQ43863.1), MoMkk1 (ELQ59117.1) and MoMps1 (XP_003712437.1) from *Magnaphorthe oryzae*; AnBck1 (CBF76548.1), AnPkcA (BAD02338.1), AnMkk1 (XP_661793.1) and AnMpk1 (AAD24428.1) from *Aspergillus nidulans*; PgBck1 (XP_003328672.2), PgMkk1 (XP_003890718.1) and PgMpk1 (XP_003335205.1) from *Puccinia graminis*; FgPck1 (XP_011328048.1), FgMkk1 (XP_011327039.1), and FgMgv1 (XP_011319273.1) from *Fusarium graminearum*; NcBck1 (XP_011395111.1), NcPck1 ([XP_960155.2](https://www.ncbi.nlm.nih.gov/protein/XP_960155.2?report=genbank&log$=protalign&blast_rank=1&RID=2WBG83FH016)), NcMkk1 (XP_957310.3) and NcMpk1 (XP_958040.2) from *Neurospora crassa*; and UmMpk1 (XP_011386664.1) from *Ustilago maydis*. The bootstrap values are indicated on the phylogenetic tree.


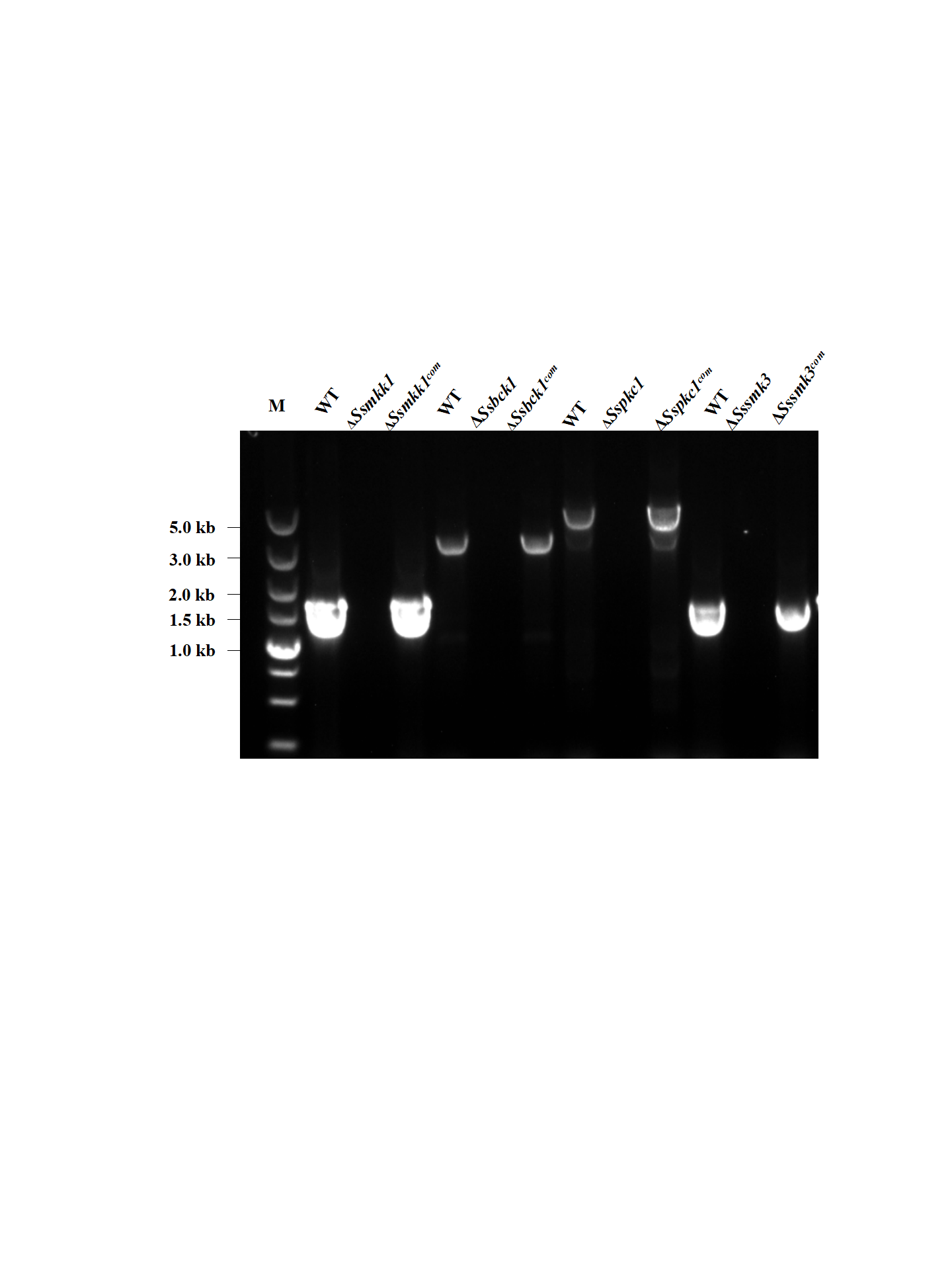


**Supplementary Figure 2.** Identifification of knockout transformants and complement transformants by PCR. Conventional PCR were performed for transformants. M, DNA molecular size marker; WT, wild type strain;lanes 2–3: putative transformants SsMkk1; lanes 5–6: putative transformants SsBck1; lanes 8-9: putative transformants SsPkc1; lanes 11-12: putative transformants SsSmk3.

**Supplementary Table 1. Primer list.**

| Primers | Sequences 5'-3' | Purpose |
| --- | --- | --- |
| SS1G_11866-F | CAGAGGATGAACCTACCGCCAAC | qPCR gene |
| SS1G_11866-R | AGTTGTTCCTTGGTGAGGTTGTCC |  |
| SS1G_01124-F | CCGAACAGCTACAGCCGATTCTC |  |
| SS1G_01124-R | TGCCAGAACAATTCACCTCCAGAC |  |
| SS1G_07715-F | CAAGCCGAAGTCCAAGCACCTG |  |
| SS1G_07715-R | TCCTTCTGTCTTGACCTCGTAGCC |  |
| SS1G_07355-F | CTCACGACGATACCTTCACAACCG |  |
| SS1G_07355-R | ATTGCCGCTCCGAGTTCACTTC |  |
| SS1G_03171-F | GCCTTCTGGAACTGGAAGCACAC |  |
| SS1G_03171-R | CGGCCTTCCACCATGTTCATCC |  |
| SS1G_12143-F | GGCAGGTCTTGAGGTTGAATCTCC |  |
| SS1G_12143-R | CTCGGCGGCAATCTTTGTTTCTTC |  |
| SS1G_06394-F | CTAGCCAGTGGAGCCATTGATGTC |  |
| SS1G_06394-R | GCGATAAGCAAAACAGCCAAGACC |  |
| SS1G_13636-F | GTCCCCAAACTCGCCACCAAAG |  |
| SS1G_13636-R | TGCCAGCGTAGCAGCAAGTTG |  |
| SS1G_04353-R | ATGCTGCGTCTATGGTGGCT |  |
| SS1G_00601-F | TGGTCATGCATTCGCTTGGC |  |
| SS1G_00601-R | CGTAGGCGCCGTAGAGAACT |  |
| SS1G_09402-F | GCGGAGTCGATGGGATTGGA |  |
| SS1G_09402-R | GCCTCAACCTTCACCCTCCT |  |
| SS1G_05959-F | CCCATGCACTCTGTTGAGCG |  |
| SS1G_05959-R | AGCGGTTGATCGGTGACCTT |  |
| SS1G_10880-F | GGCACGCATCCAACAGTTCA |  |
| SS1G_10880-R | ATGCATGGAGGCTTGGACGT |  |
| SS1G_14424-F | AAGACGGAGCGATGCTGGAA |  |
| SS1G_14424-R | CAGCTCCGCAGATGAGGACA |  |
| SS1G_12905-F | TGTCACTCACGCCTCAGCAA |  |
| SS1G_12905-R | TGGATTGGCAGCGAGGACAT |  |
| SS1G_10983-F | CCGACTCCGACTCCGACTCTG |  |
| SS1G_10983-R | TATCTCCACTGACGCTCTCCTTGG |  |
| SS1G_14026-F | TCACCACAACAACAACGACCAGAG |  |
| SS1G_14026-R | GTGGAGGGTAATCGGCAGAAGTTC |  |
| SS1G_05445-F | GTCGTGCGCCTAATCCCTCTTC |  |
| SS1G_05445-R | CTTTTGCCCTGAAGGCTTGTGC |  |
| SS1G_00059-F | AGGAGTGAAGGAGGTGTTGGTATG |  |
| SS1G_00059-R | CTTGCTATCCGCCATCCATCATC |  |
| SS1G_07136-F | CTCAGACAACCTAGCCCAACATCG |  |
| SS1G_07136-R | CTTCGCCACTGTAGCTGCCATAG |  |
| SS1G_10311-F | GGTGTCAAGGCTGCTGAAGGTG |  |
| SS1G_10311-R | GTGCTGTCAATCATTGCGGTCAAG |  |
| SS1G_01851-F | TGCCCAAGGACCTGTCACTCTC |  |
| SS1G_01851-R | GCAGGCGGTGTCCAAGTGTAAG |  |
| SS1G_13577-F | CCACCAACCTCCTCGCAAACTTC |  |
| SS1G_13577-R | TGCTGTGCTTGCTGGGAATGTC |  |
| SsFkh1-L-F | CGAGCTCGGTATGATTGAGCACAGATG | SsFkh1 mutation  and genetic complement |
| SsFkh1-L-R | GGGGTACCGAGGAAGAGCAGATGTAGAG |  |
| SsFkh1-R-F | CGGGATCCAGGAGGAACGGTATAAAGAA |  |
| SsFkh1-R-R | AACTGCAG CGCAACCAATAAACCACAT |  |
| SP-F | ATGCCATCTTCCGGTAAG |  |
| SP-R | AATAACTTACTTGCCAACCG |  |
| H-F | GAGAGCCTGACCTATTGC |  |
| H-R | AGTGTATTGACCGATTCCTT |  |
| SsFkh1-F | GTAGGAACCCAATCTTCAAAATGCCATCTTCCGGTAAGAGGG |  |
| SsFkh1-R | AGCTCCTCGCCCTTGCTCACGATGCGAGCTGAAGGTGTAACAAG |  |
| G418-F | TGTCCGGTGCCCTGAATGAACT |  |
| G418-R | GCCGCCAAGCTCTTCAGCAATAT |  |
| SS1G_10983-R | aaacAGAATGTGGACAAAGGTCGGTCCA | SsPkc1, SsBck1, SsSmk3, and SsMkk1mutation and genetic complement |
| SS1G_10983-F | agttTGGACCGACCTTTGTCCACATTCT |  |
| SS1G_14026-F | agttCATGCGATGCGCACTGTG |  |
| SS1G_14026-R | aaacAGAATGTGGACAAAGGTCGGTCCA |  |
| SS1G_05445-F | agttCTCGCTGCTATCGTACGATC |  |
| SS1G_05445-R | aaacAGAATGTGGACAAAGGTCGGTCCA |  |
| SS1G_00059-F | aaacAGAACCTTTTCACCAGTACG |  |
| SS1G_00059-R | aaacAGAATGTGGACAAAGGTCGGTCCA |  |
| SS1G_10983-F | GTAGGAACCCAATCTTCAAAATGTCAGCAACGTATATACCGCG |  |
| SS1G_10983-R | aaacAGAATGTGGACAAAGGTCGGTCCA |  |
| SS1G_14026-F | GTAGGAACCCAATCTTCAAAATGCAAAACAACGATGAGGAGGC |  |
| SS1G_14026-R | AGCTCCTCGCCCTTGCTCACCTAGTTCAAAATTAGctatgttagga |  |
| SS1G_05445-F | GTAGGAACCCAATCTTCAAAATGGCGGATCTCCAAGGAAGAA |  |
| SS1G_05445-R | AGCTCCTCGCCCTTGCTCACCTATGATCGCATAGCATCCAATC |  |
| SS1G_00059-F | GTAGGAACCCAATCTTCAAAATGTCTTCCCCAGCACCATT |  |
| SS1G_00059-R | AGCTCCTCGCCCTTGCTCACTTACCAGCCCCATACCGTGG |  |
| SS1G_07360-F | CGGAATTCATGCCATCTTCCGGTAAGAGGG | SsFkh1 and SsMkk1 BiFC |
| SS1G_07360-R | CGGGATCCTTAATAACTTACTTGCCAACCGTGGG |  |
| SS1G_00059-F | CGGAATTCATGTCTTCCCCAGCACCATT |  |
| SS1G_00059-R | CGGGATCCTTACCAGCCCCATACCGTGG |  |
| SS1G_07360-F | CGGAATTCATGCCATCTTCCGGTAAGAGGG | SsFkh1, SsMkk1 and SsSmk3 Y2H |
| SS1G_07360-R | CGGGATCCTTAATAACTTACTTGCCAACCGTGGG |  |
| SS1G_05445-F | CGGAATTCATGGCGGATCTCCAAGGAAGA |  |
| SS1G_05445-R | GGAATTCCCTATGATCGCATAGCATCCAATCCA |  |
| SS1G_00059-F | GGATCCATGTCTTCCCCAGCACCATTATTACG |  |
| SS1G_00059-R | GAATTCTTACCAGCCCCATACCGTGGC |  |
|  |  |  |
